# Neural Network–Based Clinical Prediction System for Identifying the Clinical Effects of Saffron (Crocus sativus L) Supplement Therapy on Allergic Asthma: Model Evaluation Study

**DOI:** 10.2196/17580

**Published:** 2020-07-06

**Authors:** Seyed Ahmad Hosseini, Amir Jamshidnezhad, Marzie Zilaee, Behzad Fouladi Dehaghi, Abbas Mohammadi, Seyed Mohsen Hosseini

**Affiliations:** 1 Nutrition and Metabolic Diseases Research Center Ahvaz Jundishapur University of Medical Sciences Ahvaz Iran; 2 Department of Nutrition Faculty of Allied Medical Sciences Ahvaz Jundishapur University of Medical Sciences Ahvaz Iran; 3 Department of Health Information Technology Faculty of Allied Medical Sciences Ahvaz Jundishapur University of Medical Sciences Ahvaz Iran; 4 Environmental Technologies Research Center Ahvaz Jundishapur University of Medical Sciences Ahvaz Iran; 5 Department of Occupational Health School of Public Health Ahvaz Jundishapur University of Medical Sciences Ahvaz Iran

**Keywords:** asthma, machine learning, clinical predictor system, neural networks, supplement therapy, saffron, Crocus sativus L

## Abstract

**Background:**

Asthma is commonly associated with chronic airway inflammation and is the underlying cause of over a million deaths each year. *Crocus sativus L*, commonly known as saffron, when used in the form of traditional medicines, has demonstrated anti-inflammatory effects which may be beneficial to individuals with asthma.

**Objective:**

The objective of this study was to develop a clinical prediction system using an artificial neural network to detect the effects of *C sativus L* supplements on patients with allergic asthma.

**Methods:**

A genetic algorithm–modified neural network predictor system was developed to detect the level of effectiveness of *C sativus L* using features extracted from the clinical, immunologic, hematologic, and demographic information of patients with asthma. The study included data from men (n=40) and women (n=40) individuals with mild or moderate allergic asthma from 18 to 65 years of age. The aim of the model was to estimate and predict the level of effect of *C sativus L* supplements on each asthma risk factor and to predict the level of alleviation in patients with asthma. A genetic algorithm was used to extract input features for the clinical prediction system to improve its predictive performance. Moreover, an optimization model was developed for the artificial neural network component that classifies the patients with asthma using *C sativus L* supplement therapy.

**Results:**

The best overall performance of the clinical prediction system was an accuracy greater than 99% for training and testing data. The genetic algorithm–modified neural network predicted the level of effect with high accuracy for anti–heat shock protein (anti-HSP), high sensitivity C-reactive protein (hs-CRP), forced expiratory volume in the first second of expiration (FEV_1_), forced vital capacity (FVC), the ratio of FEV_1_/FVC, and forced expiratory flow (FEF_25%-75%_) for testing data (anti-HSP: 96.5%; hs-CRP: 98.9%; FEV_1_: 98.1%; FVC: 97.5%; FEV_1_/FVC ratio: 97%; and FEF_25%-75%_: 96.7%, respectively).

**Conclusions:**

The clinical prediction system developed in this study was effective in predicting the effect of *C sativus L* supplements on patients with allergic asthma. This clinical prediction system may help clinicians to identify early on which clinical factors in asthma will improve over the course of treatment and, in doing so, help clinicians to develop effective treatment plans for patients with asthma.

## Introduction

Asthma is a heterogeneous disease and usually coincident with chronic airway inflammation. Asthma can be diagnosed based upon the patient’s history of respiratory symptoms such as wheezing, chest tightness, shortness of breath, and cough which may vary among the patient population in terms of intensity, time, and decreased expiratory airflow.

Allergic asthma is the easiest phenotype of asthma to diagnose with symptoms usually becoming apparent in childhood [1]. Globally, the prevalence of asthma in adults is estimated to be 4.3% [1]; in 2012, an estimated 300 million adults suffered from asthma, and by 2025, this figure will increase to 400 million. Moreover, the annual mortality rate of asthma is estimated to be about 250,000 [2]. In Iran, the prevalence of asthma is around 5.5% [3]. Every year, according to the World Health Organization (WHO), 15 million disability-adjusted life-years are lost because of the disease [4]. Those who develop asthma often have allergic conditions or a family history of allergic conditions such as eczema, food allergies, drug allergies, or allergic rhinitis. These patients normally respond well to inhaled corticosteroid treatments [2]. Like mast cells, different immunologic cells such as eosinophils, lymphocytes, and neutrophils have a role in the process of airway inflammation [5]. Genetic factors are among the risk factors of asthma, in addition to environmental factors such as exposure to allergens which may also exacerbate asthma symptoms and access to health care services [2].

There is a need for health care providers and governments to work collectively to improve control of asthma symptoms [2]. In traditional medicine, *Crocus sativus L,* which is more commonly known as saffron, has been used as a treatment for heart disease, depression, stress, and sleep disorders [5]. *C sativus L* possesses antioxidant [6] and anti-inflammatory properties [7]. Its active components—safranal and crocin—have demonstrated beneficial anti-inflammatory and antioxidant effects [5].

Several studies [6,8,9] have reported the effects of *C sativus L* on asthmatic patients. Zilaee et al [6] studied, in a randomized clinical trial, the effects of *C sativus L* supplements on clinical symptoms, Asthma Severity Score, blood pressure, and lipid profiles of patients with mild or moderate persistent allergic asthma. Although these studies showed aggregate effects, it was not possible to predict rare and serious effects for individuals.

Recommender systems, also known as recommendation engines, are used in online personalized predictive models and have been increasingly implemented in many areas of application to extract useful information from data; however, most of the available approaches that rely upon traditional statistical outcomes are unable to extract crucial knowledge [10,11] such as severity reduction estimates for patients with asthma [6]. Therefore, recommender systems can overlook significant effects of *C sativus L* supplements in patients with allergic asthma.

Recently, researchers have begun to estimate the effectiveness of clinical medicine using machine learning methods [12]. Machine learning techniques can be used to approximate the treatment effects of medicines [13,14]. An important application of machine learning in medicine is the development of automated risk-prediction algorithms to guide clinical care [15]. These algorithms can be used to integrate and interpret complex biomedical and health care data in scenarios where traditional statistical methods may not work [7].

To address the current limitations, a clinical prediction system based upon machine learning algorithms was developed to estimate the level of effect of *C sativus L* supplements in patients with allergic asthma. To classify clinical improvement in patients, we developed a model that determines the potential effect of *C sativus L* supplements on individual patients with asthma by extracting the factors with the greatest effect from clinical features, hematologic features, anti-inflammatory features, and Asthma Severity Score.

## Methods

### Data Description

To develop and evaluate a genetic algorithm–modified neural network model, we used a dataset [6] containing data on men (n=40) and women (n=40) with asthma who ranged in age from 18 to 65 years and who received *C sativus L* supplements. Using diagnostic criteria from the Global Initiative for Asthma, these patients had been diagnosed with mild or moderate allergic asthma by a pulmonologist in May 2017 or October 2017 and were recruited from the outpatient clinic at Imam Khomeini Hospital in Ahvaz, Iran.

Participants were asked to take one oral capsule containing 50 mg of dried *C sativus L* stigma (from the Faculty of Pharmacy at Ahvaz Jundishapur University of Medical Sciences) twice daily at 12 hour intervals. The *C sativus L* stigma was procured from Estahban, Fars Province, Iran (Herbarium code: JPS018118). The capsules contained 50 mg of dried saffron stigma and starch (as fillers). Each participant was asked to fill out questionnaires about asthma clinical symptoms and to have a sample of blood drawn. Additionally, the participants were interviewed about their socio-demographic status, job, smoking, medical background, and medication.

### Clinical Symptoms and Critical Factors

Clinical symptoms (frequency of shortness of breath during the day, frequency of shortness of breath during the night, limitations on activity, frequency of salbutamol inhaler use, and sleep problems caused by asthma symptoms) were recorded at the preintervention and postintervention. Tests for hematologic (eosinophil and basophil counts) and anti-inflammatory factors (anti-HSP: anti–heat shock protein; and hs-CRP: high sensitivity C-reactive protein), and spirometry tests (FEV_1_: forced expiratory volume in the first second of expiration; FVC: forced vital capacity; FEV_1_/FVC ratio; and FEF_25%-75%_: midphase forced expiratory flow) were conducted preintervention and postintervention. Demographic information (age, BMI, gender, weight, and smoking history) were recorded at preintervention. Table 1 lists the input parameters, their units of measurement, and their factor type.

**Table 1:** Model inputs.

Input parameters	Units of measure	Factor type
FEV_1_^a^	Liter	clinical
FVC^b^	Liter	clinical
FEV_1_/FVC	Liter	clinical
FEF_25%-75%_^c^	L/minute	clinical
Shortness of breath during the day	frequency per day	clinical
Shortness of breath during the night	frequency per day	clinical
Waking up due to asthma symptoms	frequency per day	clinical
Activity limitation	frequency per day	clinical
Salbutamol inhaler use	frequency per day	clinical
anti-HSP70^d^	ng/mL	anti-inflammatory
hs-CRP^e^	ng/mL	anti-inflammatory
Eosinophil	number/ µL	hematologic
Basophil	number/ µL	hematologic
Age	years	demographic
Smoking history	yes or no	demographic
BMI^f^	kg/m^2^	demographic
Gender	male or female	demographic
Weight	kilogram	demographic

^a^FEV_1_: forced expiratory volume in 1 s.

^b^FVC: forced vital capacity.

^c^FEF_25%-75%_: forced expiratory flow.

^d^anti-HSP: anti-heat shock protein.

^e^hs-CRP: high sensitivity C-reactive protein.

^f^BMI: body mass index.

### Genetic Algorithm–Modified Neural Network Model

#### Overview

Asthma improvement was treated as an information retrieval problem; therefore, our model was developed to determine the relationship between *C sativus L* and four types of factors: clinical, anti-inflammatory, hematological, and demographic measures. 

An artificial neural network (ANN) machine learning model [16] was developed using MATLAB (version R2018b; MathWorks Inc) software and was used to classify patients with asthma. As a preliminary processing step, selection criteria were used to identify which parameters were discriminant predictors in order to enhance algorithm performance by eliminating those that were redundant or irrelevant [15]. To select these parameters, a genetic algorithm was used to assess subsets of parameters according to each parameter’s contribution to diagnostic performance; thus, discriminant predictors were selected as factors by their effect on the diagnostic performance of the model. The genetic algorithm was applied to find the optimal structure of the ANN model. Patients with allergic asthma who received *C sativus L* supplement were classified into 5 classes.

#### Artificial Neural Network

It is possible to solve many problems using an ANN since they are capable of computing any function which is computable. These networks are mostly suitable for solving problems that can tolerate specific levels of error. ANNs are built from several layers of interconnected nodes which are called neurons. In a typical feedforward neural network, there is at least one input layer, one hidden layer, and one output layer. The number of nodes in the input layer corresponds to the number of input features; the features are analogous to the covariates or independent variables that are incorporated into a linear regression model. The output nodes represent predictions or classifications. Backpropagation algorithms in an ANN are able to train the model using teacher-based supervised learning [17]. Testing performance from backpropagation is not always satisfactory, even if training performance demonstrates high accuracy [18].

Although, many different forms of ANN exist, this study uses ANNs built from several hidden layers which are often used in nonparametric problems. Connection weights between each neighboring layer are continuously updated so that output values approach the targeted values.

**Figure 1:**
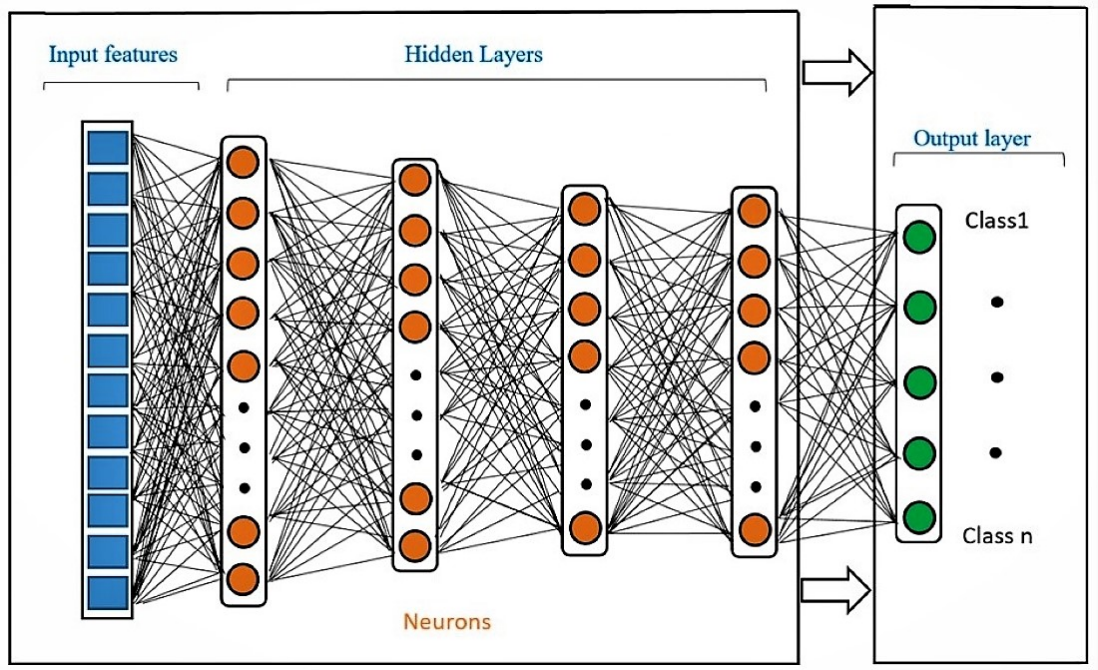
General structure of feedforward multilayer neural network.

In designing feedforward neural network topology for prediction, the components which must be considered are input layer configuration, hidden layer configuration, and output layer configuration as well as the model’s training methodology. In practice, this architecture is determined by experimentation [15]. There is a direct relationship between the input and hidden layers which operate in conjunction to apply weights to inputs and that result in new outputs (Figure 1). Eventually, the output layer classifies or predicts the outcome of the process based upon the transmitted values. The advantage of an ANN is that the network is comprised of multiple nonlinear levels which makes the ANN capable of representing highly varying functions [18]. ANNs can be used to determine complex patterns in data and may be applied in medical fields.

#### Genetic Algorithm

To find the optimal architecture of the multilayer ANN model, the input parameters, neurons, hidden layers, and learning functions were optimized using a genetic algorithm model. The genetic algorithm is a well-known optimization technique that may be categorized as an evolutionary method using biological process [19]. Genetic algorithms have been demonstrated to be reliable and robust in many medical applications [20-22]. A genetic algorithm that included chromosome reproduction, crossover, and mutation heuristic processes was implemented.

**Figure 2:**
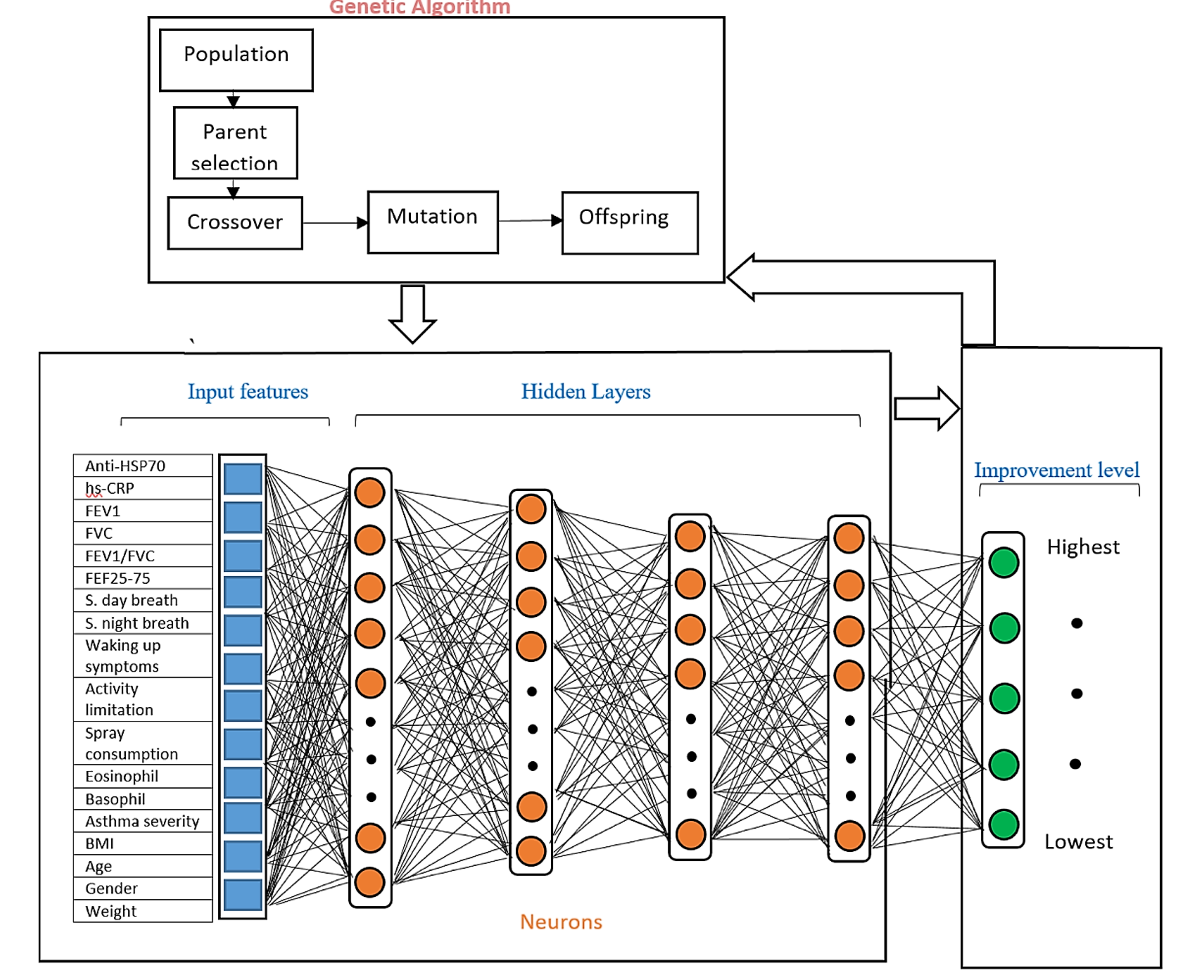
The architecture of the genetic algorithm–modified neural network system for predicting *C sativus L* supplement effects on patients with allergic asthma. Anti-HSP: anti-heat shock protein; hs-CRP: high sensitivity C-reactive protein; FEV_1_: forced expiratory volume in 1 s; FVC: forced vital capacity; FEF_25%-75%_: forced expiratory flow; BMI: body mass index.

**Figure 3:**
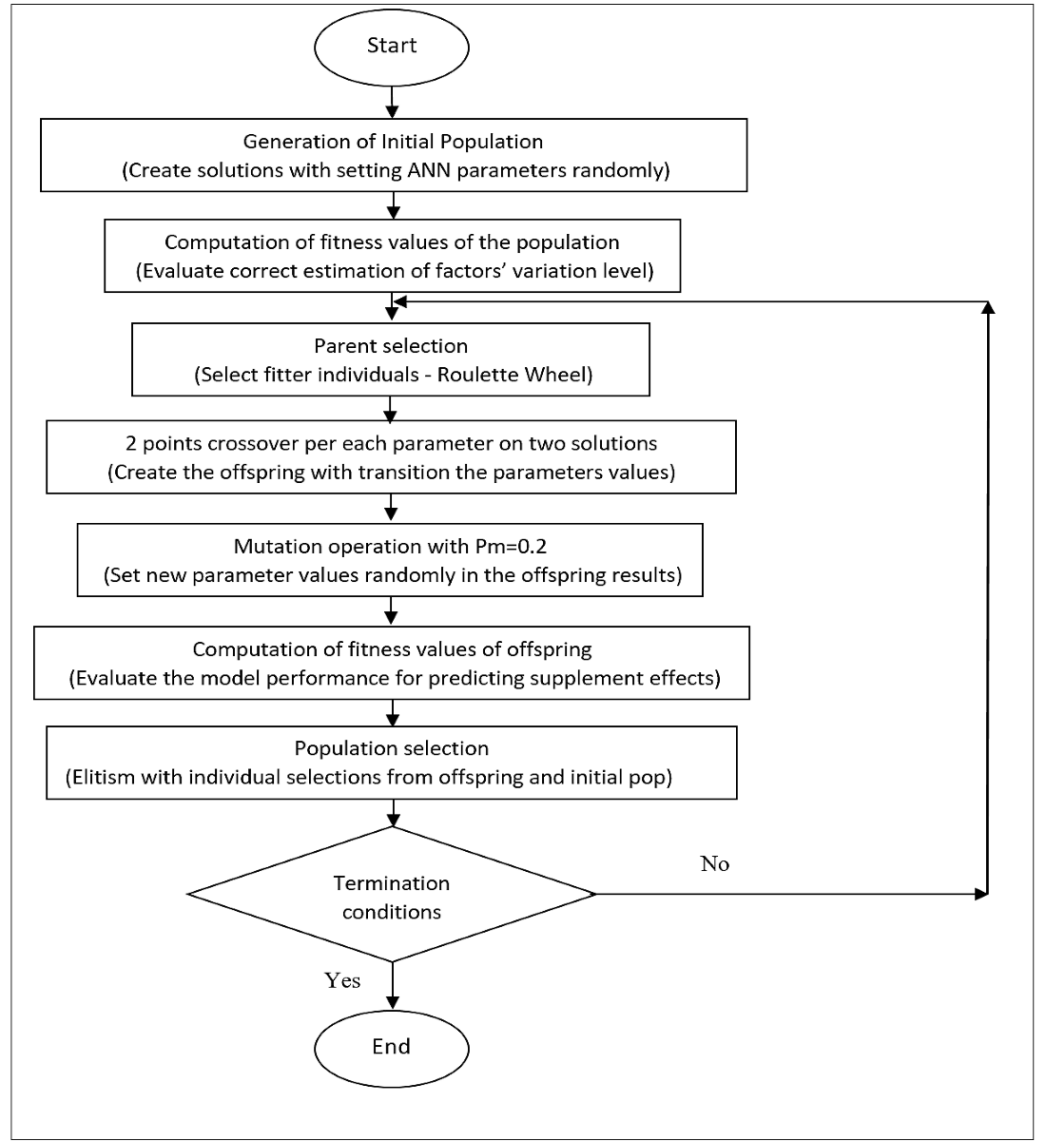
The process of the proposed genetic algorithm. ANN: artificial neural network.

The genetic algorithm–modified neural network predictive model is presented in Figure 2. The best architecture for the neural network was found by iteration of the model using the genetic algorithm that is shown in Figure 3. The genetic algorithm was used to find the most effective features to estimate the level of variation of every parameter in the patients with allergic asthma after *C sativus L* supplement therapy. Iteration was terminated when the optimal architecture was reached in terms of effective input parameters (factors) as well as hidden layers, neurons, and learning function to find the highest accuracy for prediction of effects. The accuracy rate achieved from the optimal architecture in each generation was the best fitness value. Optimization of the model occurred in two phases: (1) estimation of level of variation of each factor, separately, and (2) classification of the level of alleviation of the severity of asthma. In the first prediction process, the genetic algorithm–modified neural network system estimated the rate of variation of factors and in the second phase, the patients were classified into 5 groups from low to high level of effect of *C sativus L* supplement. The optimized neural network model predicted the level of change for each factor postintervention and classified patients into 5 groups by asthma condition.

### Performance and Validation

#### Model Performance Metrics

Mean squared error (MSE) is a commonly used error function in ANN training to evaluate the efficiency of the model. This function calculates the mean error then sends the error back to the nodes [23]. The equation for MSE and accuracy are defined as 
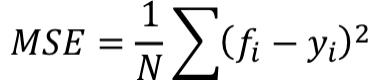
 where *N* is the total number of outputs, *f_i_* is the desired output value for each output, *i* (*i*=1, 2,…, *N*), and *y_i_* is the neural network output value for each output, *i*, and as *accuracy* = 1 – *MSE.*

#### Cross-Validation

The dataset was randomly subdivided into training (90% of available data) and testing datasets (10% of available data) using the Monte Carlo method for cross-validation (also known as repeated random subsampling) [24]. This method generated multiple random divisions of data into training and testing in 10 experiments. Furthermore, each experiment was iterated 10 times to validate of the model.

The protocol of this study was approved by the Medical Ethics Committee at Ahvaz Jundishapur University of Medical Sciences (IR.AJUMS.REC.1395.810, IR.AJUMS.REC.1398.880).

## Results

**Figure 4:**
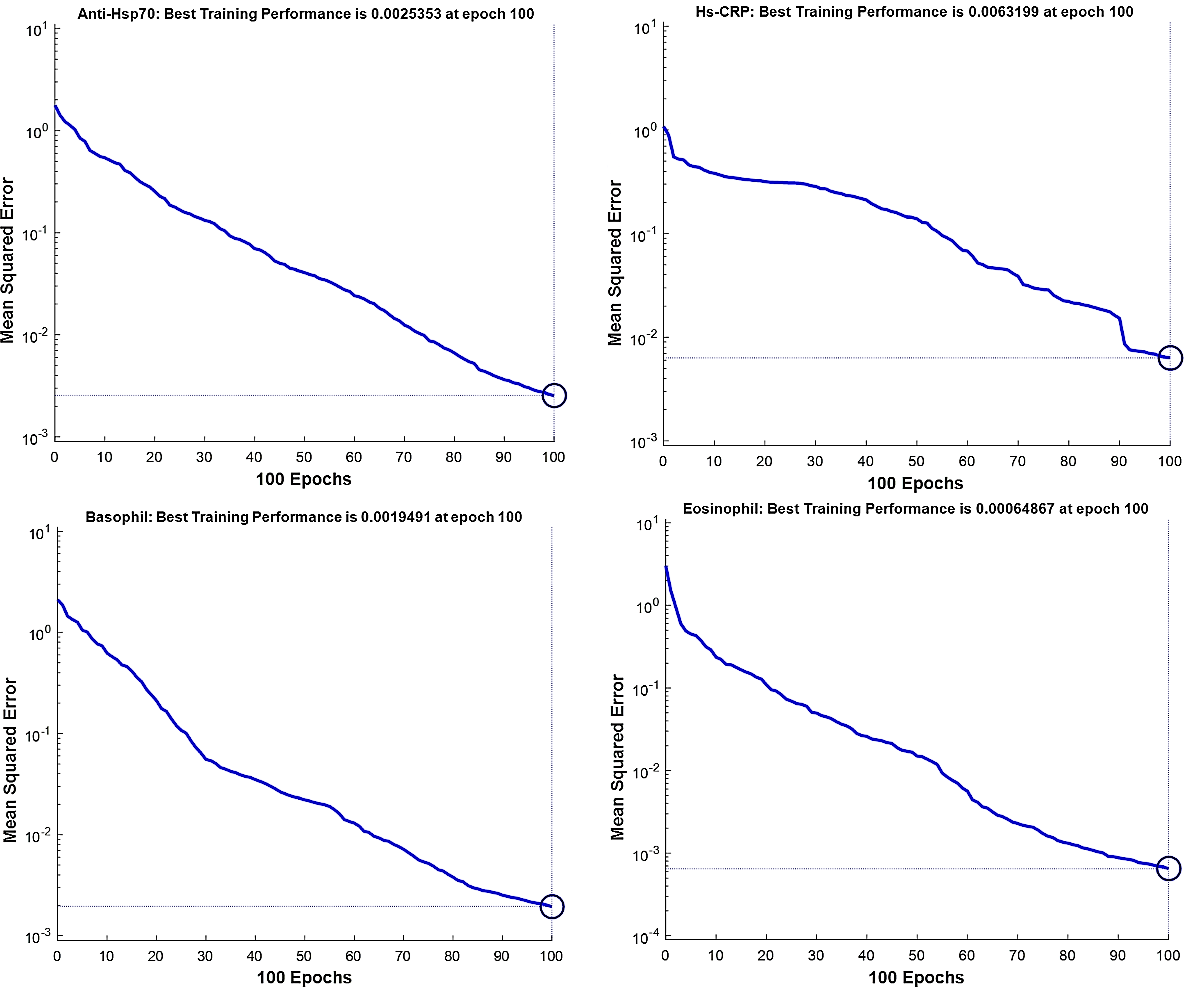
Training performance of the genetic algorithm–modified neural network for *C sativus L* supplement effects on the anti-inflammatory factors: anti-HSP70 (top left, anti-heat shock protein); hs-CRP (high sensitivity C-reactive protein, top right); and hematologic factors: basophil (bottom left); eosinophil (bottom right).

**Figure 5:**
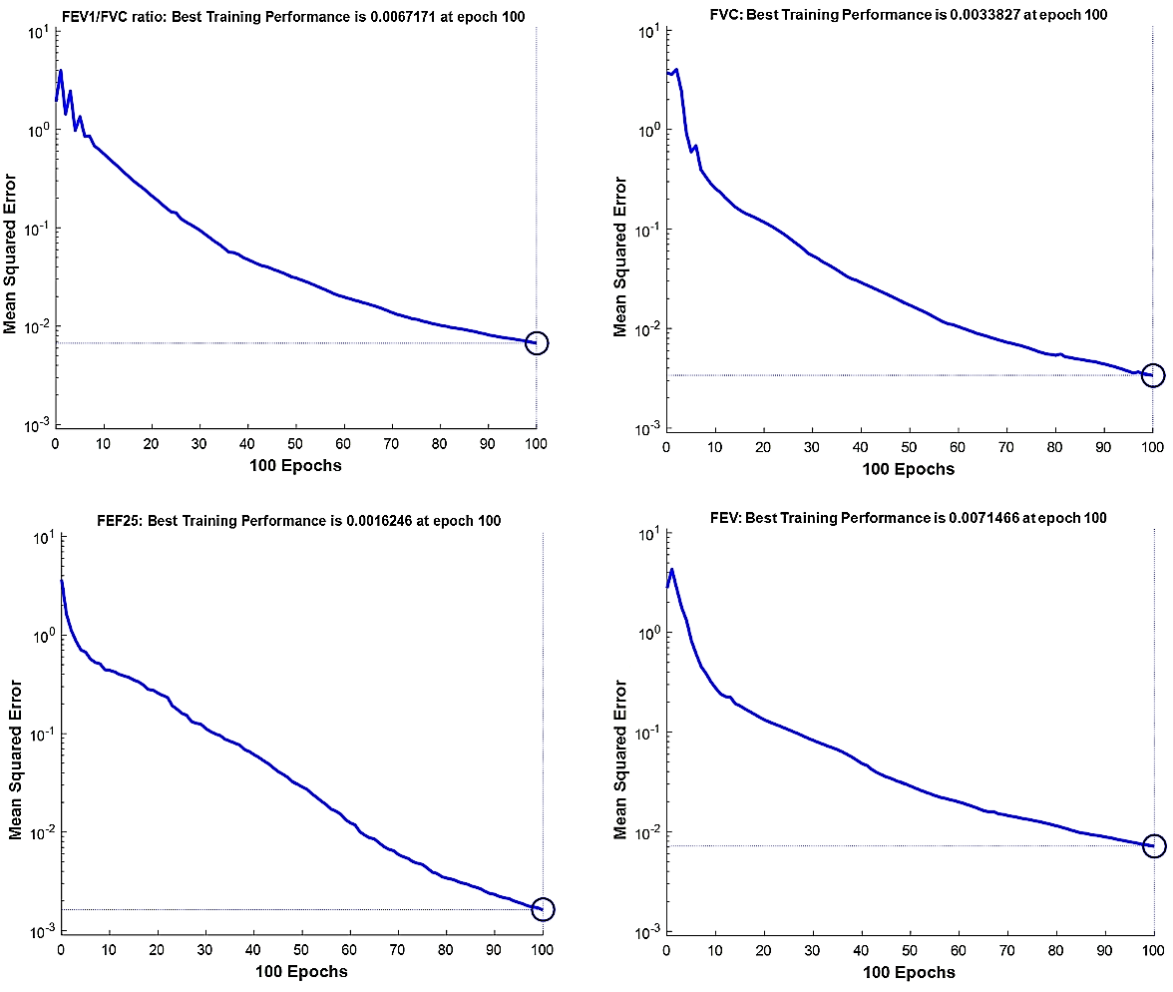
Training performance of the genetic algorithm–modified neural network for *C sativus L* supplement effects on the clinical factors: FEV_1_/FVC ratio (top left); FVC (top right); FEF-25 (forced expiratory flow, bottom left); FEV (forced expiratory volume, bottom right).

**Figure 6:**
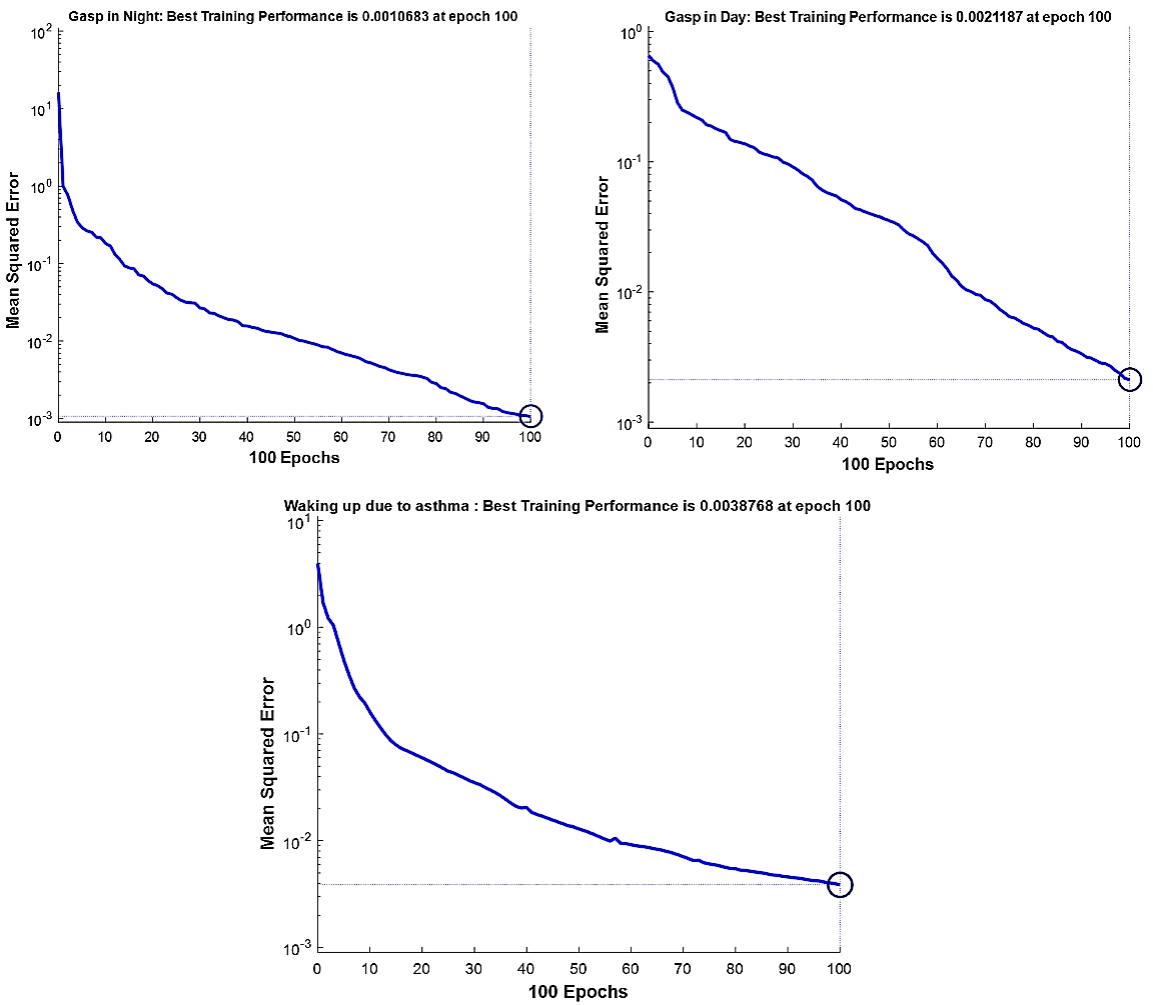
Training performance of the genetic algorithm–modified neural network for *C sativus L* supplement effects on the asthma clinical symptoms: shortness of breath in the night (top left); shortness of breath during the day (top right); waking up due to asthma symptoms (bottom).

**Figure 7:**
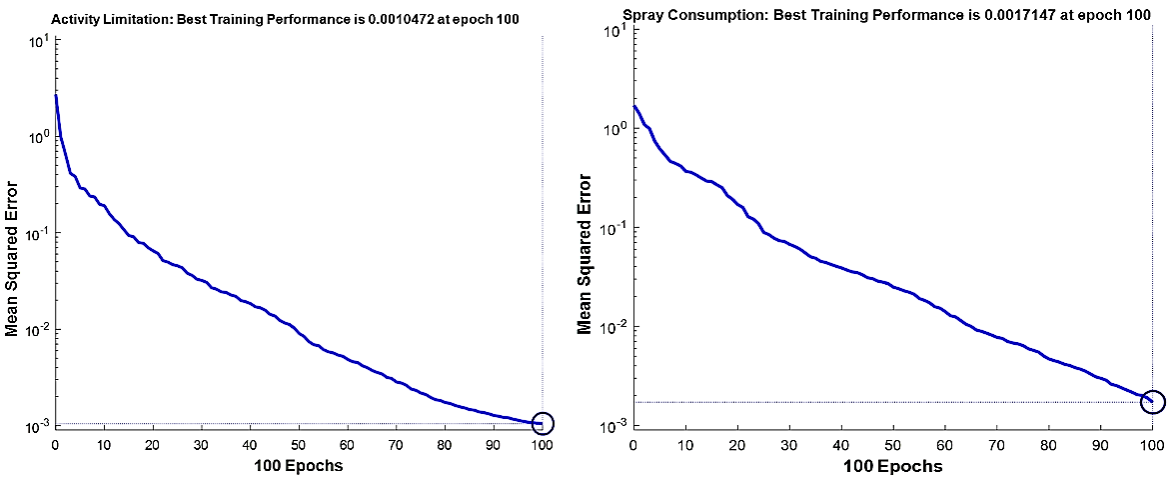
Training performance of the genetic algorithm–modified neural network for *C sativus L* supplement effects on the asthma clinical symptoms: activity limitation (left), salbutamol inhaler use (right).

The best fitness value that was achieved for each output factor is shown in Table 2. Model performance is shown in Figures 4-7. The results were achieved with 100 epochs for training process.

**Table 2:** Best training performance at epoch 100.

Factor^a^	Fitness value
FEV_1_^a^	0.007
FVC^b^	0.003
FEV_1_/FVC	0.006
FEF_25%-75%_^c^	0.002
Shortness of breath during the day	0.002
Shortness of breath during the night	0.001
Waking up due to asthma symptoms	0.004
Activity limitation	0.001
Salbutamol inhaler use	0.002
anti-HSP70^d^	0.003
hs-CRP^e^	0.006
Eosinophils	0.001
Basophils	0.002

^a^FEV_1_: forced expiratory volume in 1 s.

^b^FVC: forced vital capacity.

^c^FEF_25%-75%_: forced expiratory flow.

^d^anti-HSP: anti-heat shock protein.

^e^hs-CRP: high sensitivity C-reactive protein.

**Figure 8:**
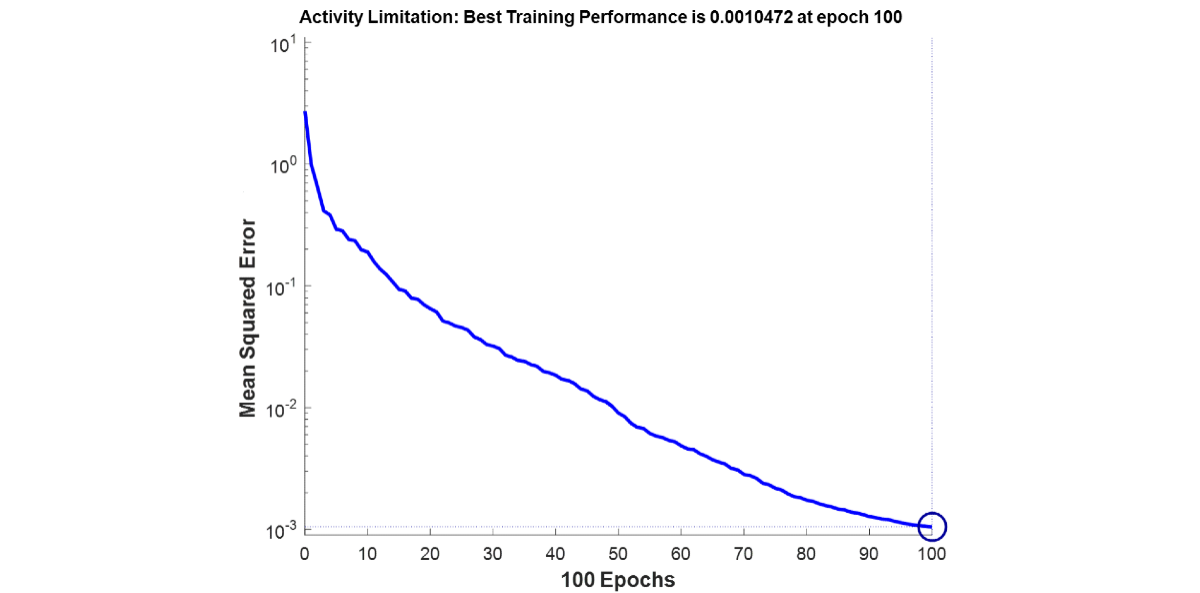
Training performance of the genetic algorithm–modified neural network for total classification of *C sativus L* supplement effects on patients with allergic asthma.

As shown in Figure 4-7, the model’s training performance reached the acceptable fitness value with the MSE lower than 5% for each factor. Furthermore, Figure 8 shows the training performance of the overall model which reached a fitness level of 3.3322 × 10^–9^. This result illustrates an accuracy greater than 99% for training. Table 3 shows the most effective factors that were found by the genetic algorithm. The selected parameters were used as input in the genetic algorithm–modified neural network to predict the effect on each clinical, hematologic, or anti-inflammatory parameter. FEV_1_/FVC ratio, waking up due to asthma symptoms, hs-CRP, eosinophil, basophil, weight, and smoking history were selected most frequently by the genetic algorithm process to estimate the level of alleviation after *C sativus L* supplement therapy. Table 4 shows the optimized architecture of the model for the test set (hidden layers, neurons, training and fitting functions), and estimated rate of risk after supplement therapy for each factor are presented. According to Table 4, the predictions for waking up due to asthma symptoms and basophil with the MSE of 0.004 and 0.045 showed the highest and lowest accuracy, respectively, among the factors in the testing phase. A pattern recognition network was selected by the genetic algorithm for most experiments (patternnet in MATLAB). Furthermore, conjugate gradient backpropagation with Polak-Ribière updates (traincgp in MATLAB) was frequently selected for training in the genetic algorithm optimization process.

According to Table 4, the best accuracy was for waking due to asthma symptoms; the model classified the patients in terms of level of alleviation with an accuracy greater than 99% (MSE=0.0004) in testing. For this prediction model, 6 factors (FEV_1_/FVC ratio, frequency of waking due to asthma symptoms, hs-CRP, eosinophil, basophil, weight, smoking condition) out of 18 input parameters were identified and used to classify the patients in five groups from low to high level of effect.

**Table 3:** Genetic algorithm feature selection results for prediction of risk factors.

Inputs selected by the genetic algorithm^a^	Factor
FEV_1_^a^/FVC^b^, FEF_25%-75%_^c^, activity limitation, salbutamol inhaler use, anti-HSP70^d^, hs-CRP^e^, eosinophil, gender, weight, smoking condition	FEV_1_
FVC, FEV_1_/FVC, activity limitation, hs-CRP, basophil, age, gender, weight	FVC
FVC, FEF_25%-75%_, shortness of breath during the day, shortness of breath during the night, activity limitation, salbutamol inhaler use, hs-CRP, basophil, age, weight, smoking condition	FEV_1_/FVC
FEV_1_, FVC, FEF_25%-75%_, salbutamol inhaler use, anti-HSP70, basophil, gender, BMI^f^	FEF_25%-75%_
FVC, FEF_25%-75%_, shortness of breath during the day, shortness of breath during the night, activity limitation, salbutamol inhaler use, basophil	shortness of breath during the day
FEV_1_, FEV_1_/FVC, FEF_25%-75%_, shortness of breath during the night, waking up due to asthma symptoms, salbutamol inhaler use, anti-HSP70, age, smoking condition	shortness of breath during the night
Shortness of breath during the day, shortness of breath during the night, waking up due to asthma symptoms, anti-HSP70, eosinophil, weight	waking up due to asthma symptoms
FEV_1_, FVC, FEV_1_/FVC, shortness of breath during the night, waking up due to asthma symptoms, activity limitation, salbutamol inhaler use, eosinophil, basophil, age, smoking condition	activity limitation
FEV_1_, shortness of breath during the night, activity limitation, salbutamol inhaler use, weight, BMI	salbutamol inhaler use
FVC, waking up due to asthma symptoms, salbutamol inhaler use, hs-CRP, eosinophil, basophil, age, gender, weight	anti-HSP70
FEV_1_, FVC, FEF_25%-75%_, shortness of breath during the day, waking up due to asthma symptoms, activity limitation, anti-HSP70, eosinophil, basophil, weight, BMI, smoking condition	hs-CRP
FEV_1_, waking up due to asthma symptoms, anti-HSP70, hs-CRP, eosinophil, basophil, BMI, age, gender, weight	eosinophil
FEV_1_, FVC, shortness of breath during the night, waking up due to asthma symptoms, salbutamol inhaler use, anti-HSP70, hs-CRP, eosinophil, basophil, gender, weight, BMI	basophil
FEV_1_/FVC, waking up due to asthma symptoms, hs-CRP, eosinophil, basophil, weight, smoking condition	total factors

^a^FEV_1_: forced expiratory volume in 1 s.

^b^FVC: forced vital capacity.

^c^FEF_25%-75%_: forced expiratory flow.

^d^anti-HSP: anti-heat shock protein.

^e^hs-CRP: high sensitivity C-reactive protein.

^f^BMI: body mass index.

**Table 4:** Genetic algorithm–modified neural network optimization results for the best testing prediction of *C sativus L* effects.

Estimated risk factor	Optimized hidden layers	Training function^a^	Fitting model^b^	MSE^c^
FEV_1_^d^	2 layers: 16 × 25 neurons	trainrp	patternnet	0.019
FVC^e^	2 layers: 26 × 25 neurons	trainrp	patternnet	0.025
FEV_1_/FVC	2 layers: 16 × 15 neurons	trainrp	patternnet	0.029
FEF_25%-75%_^f^	2 layers: 25 × 19 neurons	traincgp	patternnet	0.033
Shortness of breath during the day	2 layers: 12 × 7 neurons	traincgb	patternnet	0.010
Shortness of breath during the night	2 layers: 18 × 8 neurons	traincgb	feedforwardnet	0.005
Waking up due to asthma symptoms	2 layers: 22 × 6 neurons	trainrp	patternnet	0.004
Activity limitation	2 layers: 9 × 24 neurons	traincgf	patternnet	0.009
Salbutamol inhaler use	2 layers: 16 × 28 neurons	traincgb	patternnet	0.030
anti-HSP70^g^	2 layers: 27 × 17 neurons	traincgp	patternnet	0.035
hs-CRP^h^	2 layers: 10 × 3 neurons	trainoss	patternnet	0.012
Eosinophil	2 layers: 13 × 26 neurons	traincgb	patternnet	0.025
Basophil	2 layers: 26 × 8 neurons	traincgp	patternnet	0.045
Total	2 layers: 12 × 19 neurons	trainlm	patternnet	0.0004

^a^MATLAB training functions where trainrp is resilient backpropagation; traincgp, traincgb, and traincgf are conjugate gradient backpropagation with Polak-Ribière updates, Powell-Beale restarts, and Fletcher-Reeves updates, respectively; trainoss uses the one-step secant method; and trainlm uses Levenberg-Marquardt optimization.

^b^MATLAB fitting models where patternnet is a pattern recognition network, and feedforwardnet is a feedforward network.

^c^MSE: mean squared error.

^d^FEV_1_: forced expiratory volume in 1 s.

^e^FVC: forced vital capacity.

^f^FEF_25%-75%_: forced expiratory flow.

^g^anti-HSP: anti-heat shock protein.

^h^hs-CRP: high sensitivity C-reactive protein.

## Discussion

In this study, a clinical system to predict the effectiveness of *C sativus L* supplements in patients with allergic asthma was designed. The findings indicated that the model sufficiently estimated the level of alleviation in patients with asthma and the level of variation of asthma clinical, anti-inflammatory, and hematological factors.

Research [25] conducted on healthy individuals indicated that doses up to 400 mg per day of *C sativus L* were safe. In addition, a study [26] noted that a daily dosage of 100 mg of *C sativus L* in patients with metabolic syndrome reduced anti-HSP70 levels. Anti-HSP has been demonstrated to be a risk factor for asthma and has been correlated with asthma severity [8,26]. Another study [6] showed the beneficial effects of 100 mg *C sativus L* per day on clinical and immunologic factors as well as on symptom severity in asthma.

In a study [17], it was confirmed that it is possible to create a predictive model with machine learning algorithms that can outperform experts. Predictions can be in the form of patient classification, disease diagnosis, drug composition, and reactions to drugs. Prediction of treatment effects using machine learning have been used to develop clinical drug prediction systems [12,27]. To the best of our knowledge, our clinical prediction system is the first system that uses input features selection and classifies effectiveness of *C sativus L* supplement therapy.

The input features have an important role in the efficient implementation of prediction problems [28]. It is possible to use data mining methods to decrease the data dimensions, choose optimal features, and achieve better system precision [29]. In a study [15], partial least square regression was used to identify 9 out of 48 prognostic factors that were correlated to persistent asthma. Moreover, multilayer and probabilistic neural networks topologies were studied to find the best prediction accuracy [15]. In another study [29], data analysis was performed to select 13 effective factors to use in an ANN asthma diagnostic model [29]. We used a genetic algorithm feature selection model to find pertinent features. Genetic algorithms were recently developed to compare different feature selection methods and may be useful for feature selection when the problem has an exponential search space. The many advantages of genetic algorithms for feature selection are highlighted in the literature [30,31]. In contrast to that of other asthma diagnostic studies, the feature selection in our system is aimed to optimize the classification of asthma patients with supplement therapy into different groups in terms of the level of effect of *C sativus L* supplement.

ANN-based models have recently been used as a robust technique to classify patients with asthma, chronic obstructive pulmonary disease, or normal lung function based on measurement of lung condition and symptoms [7,32,33].

Analyses of the mean accuracies of the genetic algorithm–modified neural network predictor with feature selection included selecting effective risk factors and architecture components using an allergic asthma dataset for which the best MSE of 0.0004 was obtained. It was also clear that feature selection improved the accuracy of prediction for all asthma risk factors as well as the accuracy of the classification of patients in terms of level of alleviation.

Our study was able to show the importance prioritizing factors to predict the variation level of variation of factors in the patients with allergic asthma. Frequency of salbutamol inhaler use, frequency of waking due to asthma symptoms, and weight were the input features that were selected most often by the genetic algorithm to predict the level of variation in allergic asthma risk factors for the patients using *C sativus L* supplement therapy. Therefore, the initial value of those factors have an important role in predicting the level of alleviation of disease severity.

Several studies have listed the significant and nonsignificant effects of *C sativus L* supplements on the demographic, anthropometric, and clinical characteristics of asthma [6,8,9]. Hosseini et al [9] showed that *C sativus L* significantly affected anti-HSP and hs-CRP factor serum levels . Moreover, they showed that it increased the pulmonary volumes FEV_1_, FVC, FEV_1_/FVC, and FEF_25%-75%_ [9]. They also showed that there was no significant effect on the eosinophil count [18]. The genetic algorithm–modified neural network system in this study accurately predicted the level of *C sativus L* effects for anti-HSP (96.5%), hs-CRP (98.9%), FEV_1_ (98.1%), FVC (97.5%), FEV_1_/FVC (97%), FEF_25%-75%_ (96.7%), eosinophil (97.5%), and basophil (95.5%). Moreover, in general the patients were classified into 5 groups from low to high level of alleviation which reached accuracies of 99.9% in both training and testing experiments. FEV_1_/FVC ratio, frequency of waking up due to asthma symptoms, hs-CRP, eosinophil, basophil, weight, and smoking history were selected as effective factors to estimate the level of alleviation of asthma in patients after the *C sativus L* supplement therapy.

The results also confirmed that it is possible to rely upon the prediction process described in this paper for the early prediction of level of variation of asthma factors. This study is the first to evaluate the classification accuracy of *C sativus L* supplement effect on the patients with asthma through feature selection. Our genetic algorithm–modified neural network can predict the effects of using *C sativus L* supplement on patients with asthma. This study indicated the importance of prioritizing each factor in predicting the effect of supplement therapy on allergic asthma.

By assessing risk, this method can be viewed as an important innovation to ensure that asthma is controlled and that serious complications are avoided. This study contributes to helping doctors to identify early on which factors will improve during treatment.

It would be interesting to evaluate the genetic algorithm–modified neural network on additional groups of patients with allergic asthma or other types of asthma. In future work, development of intelligent models including heuristic algorithms for feature selection with other machine learning techniques is recommended. Clinical prediction systems can also be applied to predict or to simulate the effectiveness of other medicines on other conditions, especially where different initial patient conditions may alter the course of treatment.

## Abbreviations

ANN: artificial neural network
BMI: body mass index
FEF_25%-75%_: forced expiratory flow, midexpiratory phase
FEV_1_: forced expiratory volume in the first second of expiration
FVC: forced vital capacity
hs-CRP: high-sensitivity C-reactive protein
HSP: heat shock protein
MSE: mean squared error
WHO: World Health Organization
